# Sinonasal Angiomatous Polyp: Evaluation With 2-Phase Helical Computed Tomography

**DOI:** 10.1097/MD.0000000000001196

**Published:** 2015-07-24

**Authors:** Changwei Ding, Qiushi Wang, Qiyong Guo, Zhenhai Wang, Xiaomei Lu, Jun Zhang

**Affiliations:** From the Department of Radiology, Shengjing Hospital of China Medical University, Shenyang, P.R. China (CD, QW, QG, JZ); Department of Nasology, Shengjing Hospital of China Medical University, Shenyang, P.R. China (ZW); and CT Clinical Science, Philips Healthcare, Shenyang, China (XL).

## Abstract

Sinonasal angiomatous polyp (SAP) is a rare benign nontumorous lesion and previously considered lack of characteristic computed tomography (CT) findings. This study aimed to evaluate 2-phase helical CT for characterization of SAP.

Twelve patients with pathologically confirmed SAP underwent 2-phase helical CT preoperatively. After injection of 80 mL contrast material at a rate of 3 mL/s, early and delayed phases were obtained with delays of 30 and 120 s, respectively. The degree and pattern of enhancement were visually analyzed. The attenuation changes were also analyzed quantitatively by measuring CT values and compared with those of the internal maxillary artery (IMA).

All 12 cases showed vessel-like marked heterogeneous enhancement at both early and delayed phases. An irregular linear, nodular, and patchy enhancement pattern was found at the early phase, and enlarged and fused together, that is, progressive enhancement pattern was found at the delayed phase. There was no significant difference between the CT values of SAP and those of the IMA at the plain, arterial phase, and delayed phase (53 ± 6 Hounsfield units [HU] vs 56 ± 7 HU, 187 ± 56 HU vs 209 ± 71 HU, and 143 ± 22 HU vs 139 ± 19 HU, respectively, *P* = 0.361, 0.429, and 0.613, respectively).

Vessel-like marked heterogeneous enhancement was a characteristic CT feature of SAP, and progressive enhancement on 2-phase helical CT could further convince the diagnosis.

## INTRODUCTION

Sinonasal angiomatous polyp (SAP) is a rare benign nontumorous lesion,^1,2^ and during clinic diagnosis and imaging evaluation, it tends to be confused with a tumor, even with a malignant tumor.^1,3,4^ SAP can be cured by simple conservative surgical excision, and rarely relapses.^5^ Therefore, correct preoperative diagnosis is important for patients with SAP to avoid unnecessary extensive surgery.^3^

Computed tomography (CT) examination has become one of the preferred imaging methods for evaluating sinonasal lesions.^6^ However, only a few studies on SAP suggested that CT findings lack specificity to identify this lesion,^7–13^ even with the administration of contrast medium. Opinions vary about the contrast-enhanced CT features of the lesion, including varying degrees of patchy heterogeneous enhancement, which is usually found,^1,8^ and no enhancement.^13,14^

With faster scanning speeds and a wide range of acquisitions, helical CT enables performance of multiphase dynamic contrast-enhanced scanning without administration of additional contrast material,^15^ increasing the usefulness of CT for detection and characterization of lesions in different areas including the head and neck.^15^ However, to the best of our knowledge, there is no report about the use of multiphase helical CT for characterization of SAP.

The purpose of this study was to describe the characteristic findings of 2-phase helical CT in patients with SAP.

## MATERIALS AND METHODS

### Patients

The present study was approved by the Institutional Review Board of our hospital, and written informed consent was waived because the unlabeled uses of existing clinical materials and CT scan data analyzed in the study had no effect on the patients’ conventional diagnosis and treatment. We retrospectively reviewed 12 patients with SAP, who underwent preoperative 2-phase helical CT, and were confirmed pathologically in our hospital between December 2007 and December 2014. There were 5 male and 7 female patients, ranging in age from 18 to 62 years, with a median age of 46.5 years. The duration of symptoms before referral to our hospital ranged from 1 month to 12 years (mean, 13.6 months). The most common clinical presentations included nasal obliteration (100%, 12/12) and epistaxis (58.3%, 7/12); the other symptoms included intermittent yellow nasal discharge (41.7%, 5/12), hyposmia (25%, 3/12), maxillofacial distending pain (16.7%, 2/12), and epiphora (16.7%, 2/12). All of the patients with normal blood coagulation, had no history of craniofacial surgery and trauma, and underwent surgical removal of SAP through endoscopic sinus surgery.

### Imaging Techniques

CT scans were performed using different multislice helical CT scanners (Brilliance 64 & Brilliance iCT, Philips Healthcare, Cleveland, OH; Somatom Definition Flash, Siemens Healthcare, Erlangen, Germany). All patients underwent precontrast and 2-phase dynamic CT scanning. A bolus intravenous dose of 80 mL nonionic iodinated contrast (Iopromide, Ultravist, 300 mg I/mL, Bayer Schering Pharma, Berlin, Germany) was administered at an injection rate of 3 mL/s. Early and delayed phase scans were obtained with scanning delays of 30 and 120 s, respectively. CT scans were obtained using 120 kVp tube voltage, 200 mAs tube current, and 0.9 to 1.0 pitch factor. Direct axial and indirect coronal planes in 2- or 3-mm contiguous sections were reconstructed with soft-tissue algorithm. Bone algorithm reconstruction was also used in the precontrast CT scans. The effective radiation dose of the CT acquisition was 1.02 to 1.83 mSv, with a median of 1.56 mSv.

### Image Analysis

CT images were reviewed by 2 experienced head and neck radiologists in consensus (15 and 8 years experience, respectively). First, the original location of each lesion, that is, the maxillary sinus or nasal cavity, was confirmed according to the site of the main body of the lesion and/or the direction of the maxillary sinus medial wall displacement. The degree and enhancement pattern were visually analyzed. The degree of enhancement was subjectively assessed as follows: mild (enhancement equal to that of the masseter muscle); moderate (enhancement greater than that of the masseter muscle but less than that of nasal mucosa); marked (enhancement equal to or greater than that of the nasal mucosa); and vessel-like marked (enhancement equal to that of the adjacent small arteries or veins at the corresponding phases).

CT values (in Hounsfield units; HU) of the lesion and internal maxillary artery in each scan phase were quantitatively measured by manually drawing circular regions of interest (ROIs; 4–6 mm^2^ for the lesion and 2–3 mm^2^ for the IMA) that were not so small as to be affected by pixel variability, and not so large as to approach the edges of the lesion or vessel. During measuring the lesion's CT values, 3 to 5 ROIs were placed in the enhanced regions of early phase scans and averaged. Subsequently, the ROIs of the same size and in the same locations were placed in the precontrast and delayed phase images to obtain corresponding CT values. The IMA CT values at the arterial and delayed phases were determined in the same way. Those for precontrast scans were represented by the internal carotid artery at approximately C_1_ level, because without contrast material, the IMA was too small to be accurately defined. Time–attenuation curves of SAP and the IMA were made.

### Statistical Analysis

Wilcoxon signed-rank test was used to compare the CT values of the SAP with those of the IMA in all phases. *P* < 0.05 was considered significant.

## RESULTS

All 12 lesions appeared as expansive masses in the unilateral maxillary sinus and nasal cavity, and were considered to have arisen from the maxillary sinus. Seven of them occurred in the left side and 5 in the right side. The greatest diameters of the lesions were 3.1 to 6.4 cm, with a median of 4.3 cm.

**FIGURE 1:**
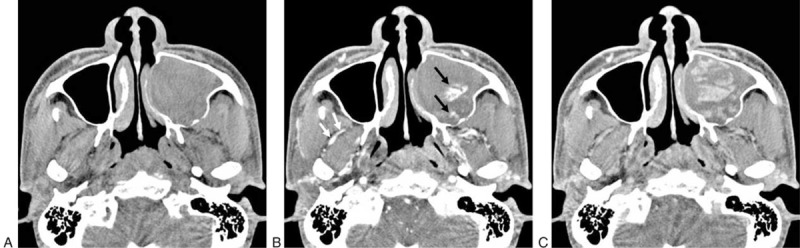
Angiomatous polyp of the maxillary sinus in a 34-year-old man. A: Axial plain CT scan shows that the left maxillary sinus is filled with a mixed-density, uneven expansile mass with remodeling and deformation of the bony wall. B: Early phase axial CT image shows multiple irregular, linear, nodular, and patchy vascular enhancement in the lesion (black arrow), of a similar degree to that of the internal maxillary artery (white arrow). C: Delayed phase axial CT image shows obvious enlargement and fusion of the enhancement region; its degree was still similar to that of the internal maxillary artery. CT = computerized tomography.

On precontrast CT images, the lesions were heterogeneous and filled the sinonasal cavity. Compared with the soft tissue of the inferior turbinate, the lesions were iso-hypo mixed-attenuated (Figure 1A).

In the visual assessment, vessel-like marked enhancement was noted in both the early phase and delayed phase CT images in all patients (Figure 1B and C). Enhancement pattern in the early phase showed as multiple irregular linear (100%, 12/12), nodular (100%, 12/12), and patchy (100%, 12/12) configuration. In the delayed phase, enhancement had a cauliflower-like appearance due to the presence of multiple strips of unenhanced septa in the lesion (100%, 12/12) (Figure 1C). Progressive enhancement was seen in all patients. This appeared as areas of enhancement that were enlarged and fused together and maintained at a high level in the delayed phase scans compared with the early phase scans (Figure 1C). The above-mentioned enhancement mainly occurred in the iso-attenuated area of the lesions. The margin of SAP was well-defined in the 120-s delayed phase CT scans due to increased contrast between the markedly enhanced lesion and nonenhanced low-density inflammation or polyp.

In the quantitative assessment, there was no significant difference between the CT values of SAP and the IMA on precontrast, early, and delayed phase CT scans (53 ± 6 vs 56 ± 7 HU, 187 ± 56 vs 209 ± 71 HU, and 143 ± 22 vs 139 ± 19 HU, respectively, *P* = 0.361, 0.429, and 0.613, respectively).

**FIGURE 2:**
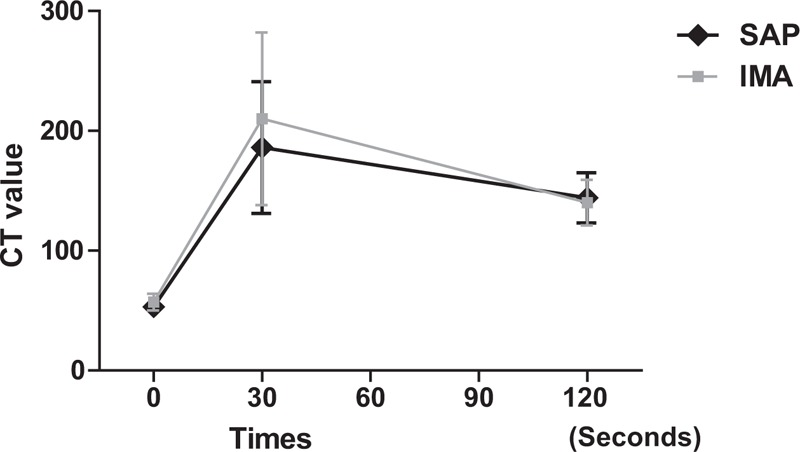
Time–density curves of the SAP and the internal maxillary artery (IMA). The degree and tendency of enhancement of SAP are similar to those of IMA. SAP = sinonasal angiomatous polyp.

Time–attenuation curves showed that the degree of enhancement of SAP was similar to that of the IMA (Figure 2).

**FIGURE 3:**
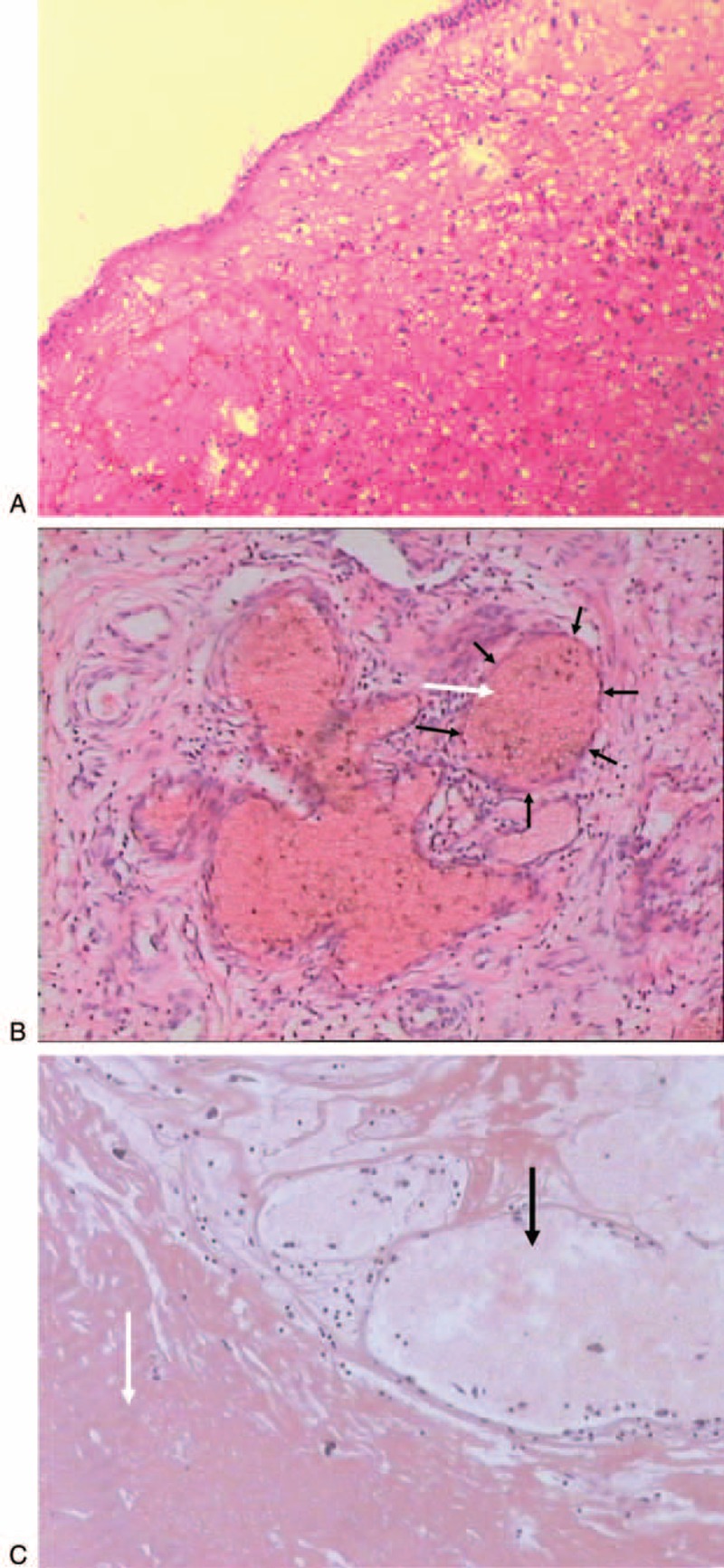
Pathological findings of angiomatous polyp of the maxillary sinus. A: Photomicrograph shows that the surface of the lesion is covered by ciliated columnar epithelium; hemorrhagic necrosis and cellulose effusion are also seen (hematoxylin and eosin [H&E] staining, ×100). B: Photomicrograph shows expansive and congested thin-walled (black arrows) blood vessels in the lesion, with edematous stroma infiltrated by many inflammatory cells (H&E staining, ×100). C: Photomicrograph shows large areas of hemorrhagic necrosis (white arrow) and expansive vessels (black arrow) in the lesion (H&E staining, ×100).

Histopathological examination showed that the surface of SAP was covered by pseudo-stratified ciliated columnar epithelium or stratified squamous epithelium with edematous stroma infiltrated by inflammatory cells (Figure 3A). The central areas of SAP showed numerous dilated thin-walled blood vessels, hemorrhage, fibrosis, and thrombus formation; scattered hemosiderin–laden macrophages and inflammatory cell infiltration were also seen (Figure 3B and C).

## DISCUSSION

SAP is described in the literature in different terms, including hematoma,^2^ organized or organizing hematoma,^1,7–11^ hematoma-like mass,^12^ hemangioma,^16–21^ angioectatic nasal polyp,^22^ and angiomatous polyp.^3,4,13,14^ These lesions all share the same clinicopathological and imaging features,^3,15^ so they are believed to describe the same lesion.^5^ In the present study, we used the term SAP.

The pathological mechanism of SAP remains poorly understood. One hypothesis is that SAP results from an organized hematoma of the maxillary sinus caused by trauma or surgery.^1,14^ Another hypothesis is that a maxillary sinus and/or nasal cavity polyp is compressed, resulting in stasis, ischemia, and necrosis of the polyp, leading to proliferation and fibrosis of new vessels.^3,23^ However, Dai et al^13^ believe that initially hemangioma develops in the maxillary sinus, which is followed by inward growth of a polyp. We agree with this proposal, because SAP often arises from the ostium of the maxillary sinus,^3^ which can readily cause obstructive inflammation, effusion, and polyps.

Clinical characteristics of SAP are varied and nonspecific. In the present study, the age range of patients was 18 to 62 years, and a wider range of 11 to 81 years has been reported.^1,3–5,13,14,22,23^ Duration of symptoms can be short or long; in our study, it was 1 month to 12 years, and in the literature, 2 weeks to 20 years.^1,3,4,13,14,22,23^ As reported previously,^1,3,8–10,12^ the most common symptoms in the present study were nasal obstruction and epistaxis. All lesions were completely resected by endoscopic sinus surgery rather than radical surgery.

CT examination reveals the bony changes and anatomical structure of the ostiomeatal complex and provides an accurate basis to determine the scope of endoscopic sinus surgery, and it has become one of the preferred imaging methods for evaluating sinonasal lesions.^6^ However, from previous reports, the CT findings for SAP are not characteristic and may be similar to those of malignant tumor. The typical appearance on precontrast CT includes a heterogeneous iso-attenuated mass that causes uneven expansile remodeling and destruction of the bony wall.^1,3,5,8,10,12,13^

Contrast-enhanced CT scans findings used to be considered uncharacteristic of SAP, and usually revealed heterogeneous enhancement with various degrees within the lesion.^1,8^ Furthermore, there are different opinions about the enhancing performance of SAP. Som et al^14^ reported, based on the findings of 5 cases, that non or minimal enhancement was found within the lesions. Similarly, Dai et al,^13^ in their series of 6 cases, reported that there was no enhancement in 1 and minimal enhancement in 5. Lee et al,^8^ based on the findings of 7 cases, found that the lesions had patchy heterogeneous enhancement, which was supposed to be typically less than that of carcinoma.^8^ In the 7 cases reported by Kim et al,^1^ contrast-enhanced CT findings revealed moderate (n = 1) or marked (n = 6) irregular nodular, papillary, or frond-like enhancement. Up to now, the conventional single-phase contrast-enhanced CT findings have varied and seem to be uncharacteristic, limiting the application of contrast-enhanced CT for diagnosis.

Present study revealed that SAP is characterized by vessel-like marked enhancement on contrast-enhanced CT scans in both early and delayed phase scans in all 12 patients. We believe that vessel-like marked enhancement is attributed to dilated neovascularization, and reveals that the lesions are composed of numerous dilated vessels. Contrast-enhanced CT findings are consistent with those of angiography. Angiography reveals feeding vessels, with or without significant enlargement, from the anterior ethmoidal and/or internal maxillary arteries to the mass, and small areas of neovascularity are also seen within the mass in the late capillary-venous phase.^14^ It is reasonable to believe that, even if single-phase contrast-enhanced CT scans are performed, vessel-like marked enhancement should also be present.

Multiphase helical CT provides a basis for the evaluation of enhancement patterns and vascularity of tumors.^15^ Two-phase CT was used in our study, and characteristic performance of progressive enhancement was observed in all 12 cases. The typical CT findings reflect the pathological and hemodynamic characteristics of SAP. Because of dilated lumina and thin-walled blood vessels, neovascular blood flow within SAP is slow. Therefore, in the early phase scans, contrast medium cannot completely fill the lesion, and merely surrounds the blood vessels, which shows as multiple irregular linear, nodular, and patchy enhancement. During the 120-s delayed phase, contrast medium progressively accumulates and persists within the dilated vascular spaces and finally fills the entire lesion, leading to enlargement and fusion of the enhanced areas. The remaining unenhanced septa in the delayed phase possibly represent thrombi or fibrous or necrotic tissue. We believe that enhancement is attributed to neovascularization, as suggested by Lee et al,^8^ and that the areas of iso-attenuation seen on unenhanced CT images represent areas of prominent vascular proliferation.^1^

Magnetic resonance imaging (MRI) is considered to be superior to CT in reflecting the internal structures and extent of SAP,^3^ and from previous findings, SAP has characteristic MRI findings, including internal heterogeneous hyper-intensity, a peripheral hypo-intense rim on T_2_ weighted image,^1,3,10^ strong nodular and patchy enhancement on postcontrast MR images, and progressive enhancement on dynamic contrast-enhanced MRI.^3^ However, contrast-enhanced CT is considered to be an excellent approach for evaluating sinus neoplasm because bone destruction is more easily seen on CT,^24^ and more lesions should be initially examined by CT. The present study suggests that 2-phase helical CT can reflect not only the progressive enhancement just like dynamic contrast-enhanced MRI but also the characteristic vessel-like enhancement of such a hyper-vascular lesion, which was not mentioned in MRI. In addition, CT is superior to MRI in displaying bony changes associated with the lesion.^1^

SAP should be differentially diagnosed from other unilateral masses in the sinonasal cavity, including inflammatory polyp, fungus ball, mucocele, inverted papilloma, and malignant tumors (such as squamous cell carcinoma, adenoid cystic carcinoma, and melanoma).^4,5,25^ Administration of contrast material is useful, because inflammatory polyp and fungus ball are not usually enhanced,^1,5^ and with mucocele, occasional peripheral enhancement may be encountered^6^ and no substantial enhancement appears.^1,3,13^ Inverted papilloma shows heterogeneous slight to moderate enhancement.^13^ Most malignant tumors also show heterogeneous enhancement, with a solid pattern of nodular enhancement.^8^ However, lack of vessel-like enhancement, rapid enhancement in the early phase and rapid washout in the delayed phase may be encountered in multiphase CT.^13^

There were several limitations in this study. First, bias arose from insufficient sample size. Second, there was no control group with other diseases to confirm the distinctive characteristics of SAP. Third, although our findings suggest that 2-phase CT may aid the diagnosis of SAP, it lacked MRI information, which is considered to show distinctive characteristics.^3^ Finally, 2-phase CT adds more radiation, although the total dose is lower than that of annual radiation exposure from natural sources (2.04 mSv). A low-dose technique should have been used to reduce radiation dose for more clinical benefit.

## CONCLUSIONS

The vessel-like marked enhancement and progressive enhancement on 2-phase helical CT scans are characteristic features of SAP, which are helpful in revealing the pathological and hemodynamic characteristics of the lesion preoperatively.
